# Anesthesia Management of Cold Agglutinin Disease in a Pregnant Patient: A Case Report

**DOI:** 10.1002/ccr3.9645

**Published:** 2024-11-29

**Authors:** Jibran Ikram, Chase Jackson, Sabry Ayad

**Affiliations:** ^1^ Department of Outcomes Research, Anesthesiology Institute Cleveland Clinic Cleveland Ohio USA

**Keywords:** autoimmune hemolytic anemia, cold agglutinin disease, multidisciplinary approach, peripartum anemia, pregnancy management

## Abstract

Cold agglutinin disease (CAD), a rare autoimmune hemolytic anemia (AIHA), is characterized by hemolysis triggered by activation of the classical complement pathway. AIHA is estimated to affect one in 100,000 people in the general population; however, its incidence in pregnant women is unclear due to the scarcity of published studies. Here, we present the case of a 37‐year‐old female (G2P1102) who presented for a repeat Cesarean section. Her peripartum course was complicated by anemia (hemoglobin 7.9 g/dL, hematocrit 29.4%) attributed to cold agglutinin disease, necessitating the transfusion of 20 units of blood during this pregnancy. She had been receiving prednisone 10 mg daily for anemia. Cold temperatures can trigger the activation of cold‐reactive antibodies, leading to red blood cells agglutination (clumping together). Preventing hypothermia reduces the likelihood of cold‐induced hemolysis, which is the primary therapeutic strategy for cold agglutinin disease. Anesthetic management for the cesarean section was accomplished via spinal anesthesia, complemented by pain control through bilateral transversus abdominis plane (TAP) blocks. This multidisciplinary approach facilitated effective pain management while considering the patient's underlying condition.

**Summary**

- This multidisciplinary collaboration in optimizing patient care is essential for managing complex medical conditions such as cold agglutinin disease (CAD) during pregnancy.
- Anesthetic management for the cesarean section was accomplished through spinal anesthesia, and pain control via bilateral transversus abdominis plane (TAP) blocks.
- Preoperative warming of these patients was of utmost importance.
- The coordination among the anesthesiology, obstetrics, and hematology teams exemplifies the importance of individualized care in addressing the unique challenges presented by CAD in the peripartum period.

## 1 Introduction

Cold agglutinin disease (CAD), a rare autoimmune hemolytic anemia (AIHA), is characterized by hemolysis triggered by activation of the classical complement pathway. AIHA is estimated to affect one in 100,000 people in the general population [1]. However, its incidence in pregnant women is unclear due to the scarcity of published studies. CAD presents unique challenges during the peripartum period. Although most patients with CAD are managed supportively, some need pharmacologic agents, while others may also require blood transfusions in the setting of severe symptomatic anemia. Pharmacologic therapy is generally offered when symptoms of anemia are clinically evident, as in our patient. Most medications used clinically target the pathogenic antibody‐producing B cells directly, with rituximab being the most administered medication [2]. We present a case of a 37‐year‐old G2P1102 female with a history of CAD and pernicious anemia who underwent delivery and required specialized multidisciplinary peripartum management.

## 2 Case History/Examination

A G2P1102 female in her 30s presented for a repeat Cesarean section. Her peripartum course was complicated by megaloblastic anemia secondary to vitamin B12 deficiency (hemoglobin 7.9 g/dL, hematocrit 29.4%) attributed to cold agglutinin disease, necessitating the transfusion of 20 units of blood during this pregnancy.

Before admission, she was placed on prednisone therapy for her cold agglutinin disease control, as advised by hematology consultants. She was initially started on a high dose of 40 mg but after several dose adjustments, hematology was able to taper the dose down to 10 mg and she remained stable until delivery. Alternative medications were discussed as viable options by maternal fetal medicine and hematology, including rituximab and sutimlimab as therapeutic considerations. However, given the lack of data during pregnancy with these medications, hematology preferred to defer rituximab and sutimlimab until after delivery, and the patient decided to try prednisone for control of her CAD.

Her other comorbidities included irritable bowel syndrome with diarrhea (IBS‐D), well controlled with dietary modification during pregnancy, and pernicious anemia, for which she received vitamin B12 1000 μg intramuscular injections.

Anesthesiology was consulted for pre‐assessment before her Cesarean section. The anesthetic plan was to keep her sufficiently warm with a forced warm air blanket throughout the hospital stay and with warmed IV fluids if necessary. As the patient had been on chronic prednisone use, the decision was made to stress dose the patient during C‐section. Additionally, the decision was to perform spinal anesthesia, complemented by a TAP block for postoperative pain management.

In the OR, the patient was prepped for a spinal block in a sterile fashion in the sitting position. The patient was monitored with standard ASA monitors, including pulse oximetry, a non‐invasive blood pressure monitor, and EKG leads. A 25G pencil tip needle was then advanced at the L3–4 interspace with clear cerebrospinal fluid noted after puncture through the dura, and 1.6 mL of 0.75% bupivacaine with 15 μg of fentanyl administered. After the procedure started, 125 mg of methylprednisolone was administered intravenously. Throughout the surgery, the patient had a MAP in the 70s, with a heart rate in the 70s. Normal sinus rhythm was noted on the monitor throughout the procedure as well. Following the delivery of the baby, bilateral TAP blocks were performed with bupivacaine 100 mg and liposomal bupivacaine 266 mg. The patient was taken to PACU hemodynamically stable. No significant anesthesia events were noted. Postoperatively, the patient's hemoglobin dropped from 9.0 to 7.9, and on post‐op day 1, the patient received 2 additional units of packed red blood cells with a blood warmer with repeat hemoglobin 10.4. The patient met appropriate postpartum milestones and was discharged home in stable condition on post‐op day 3. The patient was instructed to continue taking prednisone 20 mg for 2 days postpartum and then decrease to 10 mg daily per hematology.

## 3 Methods (Differential Diagnosis, Investigations, and Treatment)

The patient's anemia was attributed to cold agglutinin disease, supported by prior diagnostic tests including cold agglutinin titers and a positive Coombs test. Hematology monitored her cold agglutinin levels, hemoglobin, and hematocrit throughout the pregnancy. The patient was managed with blood transfusions, corticosteroid therapy (prednisone), and vitamin B12 injections. During the cesarean section, spinal anesthesia with bilateral transversus abdominis plane (TAP) blocks for pain control was administered. Preoperative warming measures were employed to prevent cold‐induced hemolysis, and she was stress‐dosed with 125 mg of methylprednisolone intraoperatively.

## 4 Conclusion and Results (Outcome and Follow‐Up)

The patient's peripartum course was successfully managed through a multidisciplinary approach involving obstetrics, anesthesiology, and hematology teams. CAD presents unique challenges in the peripartum period, necessitating prompt recognition and management. While traditional therapies such as blood transfusions and corticosteroids remain cornerstone treatments, novel agents like sutimlimab offer promising alternatives with improved efficacy and safety profiles. Individualized therapy is essential, considering the patient's clinical presentation, treatment response, and potential adverse effects. By tailoring management strategies, we can achieve better outcomes while minimizing the burden of disease and treatment‐related complications for patients with cold agglutinin disease. Postoperative hemoglobin improved to 10.4 g/dL. Hematology recommended continuing prednisone postpartum and reassessing the need for alternative therapies such as rituximab or sutimlimab.

## 5 Discussion

CAD is characterized by the presence of cold‐reactive autoantibodies, resulting in hemolytic anemia in cold temperatures. Its prevalence is estimated to range from 5 to 20 cases per million, with an annual incidence of 0.5–1.9 cases per million, exhibiting significant climatic and seasonal fluctuations [3]. CAD falls under the category of AIHAs, where red blood cells (RBCs) are destroyed through antibody‐mediated hemolysis, intravascularly and extra vascularly [4]. AIHAs are further classified into cold and warm AIHA, based on the temperature at which antigen–antibody reactions occur. In CAD, immunoglobulin M (IgM) antibodies form against antigens on RBCs in most cases, the carbohydrate antigen I, due to cold temperatures, typically around 3°C–4°C, triggering the formation of complement‐mediated factors [4]. Primary CAD occurs when agglutination arises from an autoimmune process linked to an underlying lymphoproliferative condition, whereas secondary CAD results from infectious conditions leading to the development of autoantibodies. Secondary CAD causes include drugs, lymphoproliferative disorders, and autoimmune diseases that promote the formation of antibodies against RBC antigens. Additionally, it is important to note that recent evidence categorizes CAD as a distinct clinicopathological entity characterized by a clonal, low‐grade lymphoproliferative disorder in the bone marrow, leading to the production of monoclonal autoantibodies, typically of the IgM class [3]. For instance, the World Health Organization (WHO) recognized it as a distinct lymphoid neoplasm in its classification of hematolymphoid tumors [5].

The diagnostic criteria for CAD include the presence of chronic hemolysis, a strongly positive monospecific direct antiglobulin test for C3d, a cold agglutinin titer of ≥ 64, and the absence of clinically or radiologically evident lymphoma or relevant infections [2, 5]. Patients with AIHA may present with signs and symptoms of hemolytic anemia, such as pallor, lightheadedness, exertional dyspnea, palpitations, dark‐colored urine, weakness, and fatigue. Severe anemia may necessitate blood transfusions, as observed in our patient. Diagnosis of CAD involves clinical and laboratory evaluations, including direct Coombs testing, antibody titers, and peripheral blood smears, to differentiate between the various types of AIHA. This patient presented with severe peripartum anemia necessitating blood transfusions. Although she had previously tested positive for antibody screening, a formal diagnosis of CAD was made only after her conception, during her second pregnancy, when confirmatory tests were performed. She was not tested for Mononucleosis or Mycoplasma at the time. She exhibited low Hgb levels, elevated lactate dehydrogenase, and positive cold agglutinin titers. While some cases of CAD manifest with multiple different complaints, some having acrocyanosis [4], our patient presented with severe symptomatic anemia. Some patients may present with mixed‐type AIHA or atypical laboratory findings, complicating the diagnosis.

Management of CAD is tailored to the patient's clinical presentation and underlying cause under the supervision of a hematologist. Recently there has been outstanding advancement in the treatment and management of CAD. Treatment aims to stabilize the patient, primarily avoiding cold exposure and supportive measures such as blood transfusions and corticosteroids while addressing any underlying conditions. In this case, the patient responded well to PRBC transfusions and prednisone therapy, improving Hgb levels. Prednisone is not usually prescribed for CAD, as this condition does not normally respond to glucocorticoid therapy but is indicated in special cases with low dose titers of CAD‐related antibodies < 1:32 [7]. Our patient demonstrated < 1:32 dilutions with her cold agglutin testing before her pregnancy began. Rituximab monotherapy has demonstrated response rates of around 50%, with few patients achieving complete responses, and a median response duration of 11–12 months [6]. Combining rituximab with bendamustine or fludarabine can enhance these results, although this may increase the risk of short‐term toxicity and raise concerns about potential long‐term adverse effects [6]. Other therapeutic options for CAD include novel agents such as sutimlimab, a monoclonal antibody targeting the C1s factor of the complement system, approved by the FDA in 2022. Sutimlimab inhibits complement pathways, alleviating complement‐mediated hemolysis and improving red blood cell indices. It has demonstrated high response rates and a favorable side effect profile, particularly in patients with acutely severe disease who may require temporizing measures. Individualized therapy is crucial, considering patient‐specific factors and treatment responses. In our patient, steroids effectively controlled the disease, obviating the need for chemoimmunotherapy, complement inhibitors, and invasive procedures like plasmapheresis. Tailoring management strategies can minimize side effects and optimize outcomes.

## Author Contributions

**Jibran Ikram:** conceptualization, methodology, project administration, validation, visualization, writing – original draft, writing – review and editing. **Chase Jackson:** conceptualization, methodology, validation, visualization, writing – original draft, writing – review and editing. **Sabry Ayad:** conceptualization, investigation, methodology, supervision, validation, visualization, writing – original draft, writing – review and editing.

## Conflicts of Interest

The authors declare no conflicts of interest.

## Consent

The authors certify that they have obtained all appropriate written patient consent forms. In the form, the patient(s) has/have given his/her/their consent for his/her/their images and other clinical information to be reported in the journal. The patients understand that their names and initials will not be published, and due efforts will be made to conceal their identity.

## Data Availability

The data that support the findings of this study are available on request from the corresponding author. The data are not publicly available due to privacy or ethical restrictions.
